# High refractive index in low metal content nanoplasmonic surfaces from self-assembled block copolymer thin films†

**DOI:** 10.1039/c8na00239h

**Published:** 2018-11-22

**Authors:** Alberto Alvarez-Fernandez, Karim Aissou, Gilles Pécastaings, Georges Hadziioannou, Guillaume Fleury, Virginie Ponsinet

**Affiliations:** Laboratoire de Chimie des Polymères Organiques (LCPO), CNRS UMR 5629, ENSCPB, Université de Bordeaux 16 Avenue Pey-Berland F-33607 Pessac Cedex France gfleury@enscbp.fr; Univ. Bordeaux, CNRS, Centre de Recherche Paul Pascal (CRPP) UMR 5031 33600 Pessac France ponsinet@crpp-bordeaux.cnrs.fr

## Abstract

Materials with a high and tunable refractive index are attractive for nanophotonic applications. In this contribution, we propose a straightforward fabrication technique of high-refractive index surfaces based on self-assembled nanostructured block copolymer thin films. The selective and customizable metal incorporation within out-of-plane polymer lamellae produces azimuthally isotropic metallic nanostructures of defined geometries, which were analysed using microscopy and small-angle X-ray scattering techniques. Variable-angle spectroscopic ellipsometry was used to relate the geometrical parameters of the metallic features and the resulting refractive index of the patterned surfaces. In particular, nanostructured gold patterns with a high degree of homogeneity and a gold content as low as 16 vol% reach a refractive index value of more than 3 in the visible domain. Our study thus demonstrates a new route for the preparation of high refractive index surfaces with a low metal content for optical applications.

## Abstract 2

Block copolymer-templated gold nanostructured surfaces of high refractive index due to controlled plasmonic nanoparticle shape.

## Graphical abstract

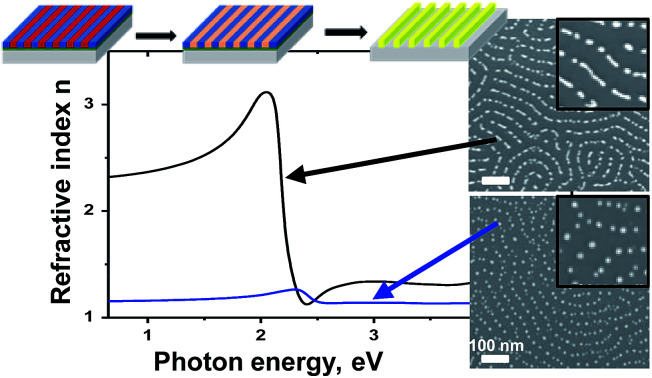

## Introduction

The control of light propagation and refraction in thin layers of materials is crucial for the development of optical and optoelectronic devices for high-resolution imaging and lithography, enhanced photovoltaic and Raman substrates, or optical communication.^1–6^ Such control can be obtained by nanostructured metal/dielectric composite thin films, due to their localized surface plasmon resonances (LSPR).^7–10^ Of particular interest is the design of high refractive index surfaces, which are crucial for a variety of applications, ranging from optoelectronics to photolithography.^11,12^ Indeed a threshold value close to 2 in the visible spectrum is commonly accepted for naturally available transparent materials.^13–15^ Optical metasurfaces with LSPR into the visible wavelength range have the potential to overcome these limitations. However, LSPR properties of simple low-density metal nanoparticle (NP) arrays induce weak optical index modulations, while dense ordered arrays, supporting plasmonic couplings or lattice modes^16^ with strong refraction effects, are usually impaired by high absorption and in-plane anisotropy, and most of the time require complex fabrication processes *via* top-down approaches of low scalability.^17–19^ To circumvolve this technological issue, bottom-up approaches including colloidal assembly,^9,11,20–22^ micellar-induced assembly,^23–26^ or self-assembly of block copolymers^27–30^ (BCPs) have been successfully introduced as alternative methodologies since they give access to NPs arrays of desired shape, characteristic size and symmetry. In particular, the selective hybridization of polymeric domains with metallic species within BCP thin films is known as a fabrication methodology for visible-range metamaterials.^31–34^ Indeed, the nanodomains generated from the microphase separation can be used as scaffolds to subsequently produce the metallic NPs arrays either by an *ex situ*^35–39^ or an *in situ*^26,34,40–43^ methodology. While the *ex situ* method is an interesting approach to introduce multi-metallic NPs into self-assembled BCP scaffolds, this route often induces an ill-defined positional distribution of the NPs due to their possibly disruptive effect on the BCP microphase separation process.^44^ The *in situ* route provides an enhanced control over the NPs positioning through the selective binding of metallic salts into pre-assembled nanostructured BCP thin films. A subsequent reductive process induced either by UV irradiation, plasma or redox chemistry,^34,45,46^ yields well-ordered metallic feature arrays, in which the orientational and positional order of the NPs can be further improved by directed self-assembly methodologies.^27,30,47^ This practical approach has been widely used to generate NPs arrays from cylinder-forming BCP thin films,^42,43,48,49^ but such nanostructured surfaces remain in the low density regime because the inter-particle distance, *p*, and their diameter, *d*, are strongly correlated following the relationship:1
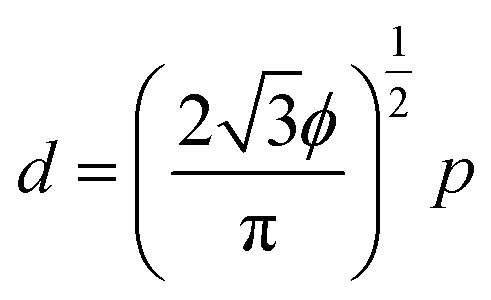
where *ϕ* is the volume fraction of the minority block, and is restricted to values close to 0.3 for the cylinder-forming BCPs. Also, such systems offer little options for variation of the shape of the produced NPs, unlike the lamellar nanostructures, as shown in the following.

Herein, we propose a straightforward fabrication technique of high refractive index surfaces based on the hybridization of a lamellar-forming poly(styrene)-*b*-poly(2-vinylpyridine) (PS-*b*-P2VP) thin film with metallic gold salts, which are preferentially incorporated within the out-of-plane P2VP lamellae. The precise control of the gold loading in the BCP nanostructure combined with the little configurationally restrictive lamellar phase yields an extended range of partially ordered metallic feature arrays with sub-visible wavelength characteristic size, *i.e.* from the formation of discrete NPs to continuous metallic lines, with a high refractive index relying on nanoparticle shape rather than plasmonic couplings. We first describe the preparation of the gold loaded nanostructured BCP thin films through the use of the selective affinity of the metallic precursors with the P2VP domains. The structural characterization of these decorated surfaces allows identifying the key parameters for the fine tuning of the final structure. Finally, variable-angle spectroscopic ellipsometry measurements are used to probe the optical properties of the gold decorated surfaces in order to retrieve the refractive index as a function of the density, shape and spatial arrangements of the NPs.

## Experimental

### Materials

The PS-*b*-P2VP, with *M*_n_ (PS) = 102.0 kg mol^−1^ and *M*_n_ (P2VP) = 97.0 kg mol^−1^, was purchased from Polymer Source Inc. and used as received. Acid tetrachloroauric (HAuCl_4_) (99.999% trace metals basis) and propylene glycol monomethyl ether acetate (PGMEA) (Reagent Plus, ≥99.5%) were purchased from Sigma-Aldrich and Merck respectively, and used without further purification. (100) silicon wafers were purchased from Si-Mat silicon materials and cut to appropriate dimensions (approximately 1 × 1 cm^2^). Silicon substrates were chemically modified by spin coating (2000 rpm) a 1.5 wt% solution of a polystyrene-*stat*-poly(methyl methacrylate) (PS-*stat*-PMMA) copolymer (*ϕ*_PS_ = 0.63) in PGMEA with a subsequent annealing at 240 °C for 10 minutes to promote the grafting, then rinsed several times with pure PGMEA to remove the non-grafted chains and dried under N_2_ flow. 100 nm thick silicon nitride membranes prepared for TEM studies were purchased from Agar Scientific.

### Self-assembly of PS-*b*-P2VP thin films and impregnation and reduction processes

Out-of-plane PS-*b*-P2VP lamellae were obtained directly by spin-coating of a 1 wt% BCP solution in PGMEA onto the modified Si wafers. 1 wt% solutions of HAuCl_4_ in EtOH or milli-Q water were used to impregnate the BCP thin films either by spin-coating (2000 rpm) or immersion of the sample in the solution. The samples were exposed to an O_2_ reactive ion etching (RIE) plasma in a PE-100 chamber (plasma conditions: O_2_ (10 sccm), 60 W, 60 s), in order to remove the BCP template and reduce the gold salts.

### Atomic force microscopy (AFM)

AFM (Dimension Fast Scan, Bruker) was used in tapping mode to characterize the surface morphology of the different films. Silicon cantilevers (Fastscan-A) with a nominal tip radius of 5 nm and a spring constant about 18 N m^−1^ were used. The resonance frequency of the cantilevers was about 1400 kHz.

Kelvin Probe Force Microscopy (KPFM) experiments were performed in ambient conditions using a commercial AFM (Dimension Icon, Bruker) in frequency modulation mode (FM-Kelvin Probe Force Microscopy) with highly doped Si probes (PFQNE-AL, Bruker). For KPFM experiments, AC voltages of 5 V were applied to the sample. In FM-KPFM, the contact potential difference (CPD) is measured simultaneously to the surface imaging. KPFM images were analyzed with Nanoscope analysis software (V1.8, Bruker).

### Scanning electron microscopy (SEM)

A Jeol 7800-E Prime SEM was used at low acceleration voltage (1 kV) in the super high resolution gentle beam (GBSH) mode to characterize the nanostructure of the patterned surfaces.

### X-ray photoelectron spectroscopy (XPS)

XPS analyses were carried out using a Thermo Fisher Scientific K-Alpha spectrometer with a monochromatic Al Kα source (*E* = 1486.6 eV). The X-ray spot size was 200 μm. The spectrometer was calibrated with monocrystalline gold and silver foils. Surveys were acquired at a 200 eV pass energy and the high resolution spectra were acquired with a pass energy of 40 eV. Thermo Scientific Avantage software was used for fitting and quantification.

### Grazing-incidence small-angle X-ray scattering

GISAXS experiments were performed on the Dutch Belgian Beamline at the European Synchrotron Radiation Facility (ESRF) station BM26B in Grenoble (12 keV). The incidence angle was set in the range of 0.12–0.19°, which is between the critical angles of the PS-*b*-P2VP film and the silicon substrate. The beam illuminates the samples with a typical footprint of 150 mm^2^. 2D scattering patterns were collected with a PILATUS 1M Dectris detector and the sample-to-detector distance was set to 3060 mm. The beam center position and the angular range were calibrated using a silver behenate standard sample. GISAXS patterns were reduced using a home-made Matlab-based code. Intensity cuts along the horizontal *q*_*y*_ were extracted from the GISAXS patterns after normalization for the incident photons and the exposure time by averaging the intensity of 5 adjacent pixel arrays, where *q*_*y*_ = 2π/*λ*[sin(2*θ*_f_)cos(*α*_f_)] and *q*_*z*_ = 2π/*λ*[sin(*α*_f_) + sin(*α*_i_)] are the modulus of the scattering vectors in the direction parallel and perpendicular to the substrate plane and *α*_i_, 2*θ*_f_ and *α*_f_ are the incident and scattering angles in the horizontal and vertical directions, respectively.

### Variable angle spectroscopic ellipsometry

The optical study of the films deposited on silicon-wafers was performed using variable angle spectroscopic ellipsometry (VASE) in reflection with a phase modulated spectroscopic ellipsometer (UVISEL, from Horiba Scientific) on the spectral range [0.6–4.8 eV or 258–2000 nm]. We used the UVISEL II (*A* = 45°; *M* = 0°) configuration, where *A* and *M* denote the azimuthal orientations of the input polarizer and the photoelastic modulator, respectively, with respect to the plane of incidence. Three values of the incidence angle *θ*_0_ = 50°, 60° and 70° were used and analysed simultaneously. The spot size was 1 mm and the measured data were checked to be similar at three different locations on the samples. We acquired the ellipsometric quantities *I*_s_ = sin (2*ψ*)sin(*Δ*) and *I*_c_ = sin(2*ψ*)cos(*Δ*), where *ψ* and *Δ* are the two ellipsometric angles, defined by the ellipsometric ratio *ρ* = *r*_p_/*r*_s_ = tan(*ψ*)e^i*Δ*^, with *r*_p_ and *r*_s_ being the complex reflection coefficients of the amplitude of the *p*-polarized (*i.e.* in the plane of incidence) and the *s*-polarized (perpendicular to the plane of incidence) waves respectively. The values of *I*_s_ and *I*_c_*vs.* wavelength were then analysed using the DeltaPsi2 software from Horiba Scientific. Spectroscopic ellipsometry data measured on the bare silicon substrate were analysed using the Si and SiO_2_ tabulated dielectric functions and yielded a thickness value (2.0 nm) for the native silica layer on the surface, which was fixed in the further analysis. The goodness of the fits was assessed by the value of the usual *χ*^2^ parameter.

## Results and discussion

**Fig. 1:**
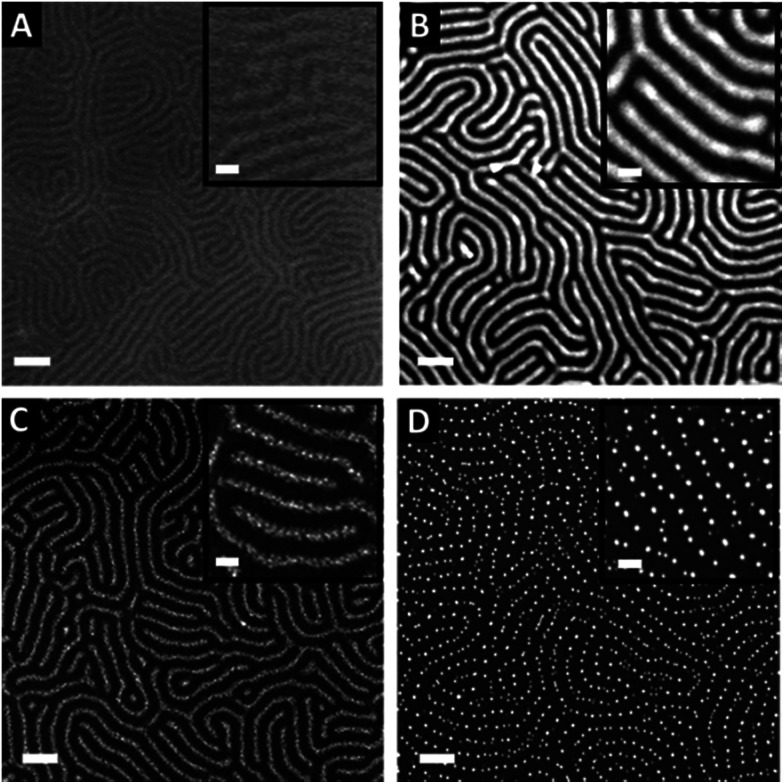
Top-view SEM images showing the different steps of the fabrication process of gold decorated surfaces templated from nanostructured BCP thin films. (A) Out-of-plane lamellae are spontaneously formed after the PS-*b*-P2VP thin film deposition. (B) Selective impregnation of the P2VP domains by spin-coating of a 1 wt% HAuCl_4_ solution on top of the BCP thin film. (C) and (D) Formation of the gold NPs obtained after reduction of the impregnated gold salts through an O_2_ plasma chemistry (*C* = 10 s, *D* = 60 s). Scale bars = 100 nm.

**Fig. 2:**
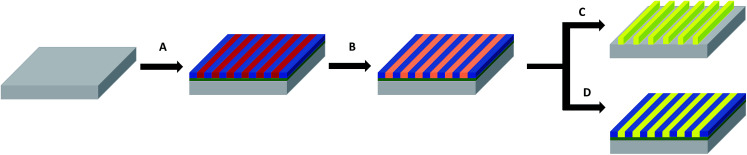
Schematic illustration of the fabrication of the gold decorated surfaces derived from nanostructured BCP thin films. (A) Formation of an out-of-plane lamellar structure obtained directly after spin-coating. (B) Selective impregnation of the P2VP domains by either spin-coating or immersion of a HAuCl_4_ solution. (C) Formation of the nanostructured gold surfaces by reduction of the impregnated gold salts through plasma RIE. (D) Formation of the metal/polymer composite surfaces by reduction of the impregnated gold salts through UV light irradiation.

The nearly symmetric PS-*b*-P2VP system used in this study has a molecular weight of 199 kg mol^−1^ and a PS volume fraction *ϕ*_PS_ of 0.53. The SAXS powder pattern obtained from a thermally-annealed thick sample (see Fig. S1A†) was indexed to a lamellar morphology with a periodicity, *L*_0_ of 63 nm. This result is in accordance with the lamellar morphology (*L*_0_ = 64 nm) formed by as-cast PS-*b*-P2VP thin films (thickness, *t* = 32 nm) deposited from a 1 wt% BCP solution in PGMEA, for which an out-of-plane orientation with symmetrical PS (dark) and P2VP (light) layers is observed, as shown on the SEM image displayed in Fig. 1A as well as on the AFM topographical view presented in Fig. S2A.† Interestingly, there is no defined azimuthal orientation, which is consistent with the characteristic fingerprint patterns formed by untemplated lamellar-forming BCP thin films. The gold decorated surfaces were subsequently produced by the hybridization of the P2VP lamellae with gold salts following the methodology sketched in Fig. 2. The selective gold incorporation into the P2VP domains is insured by the Brønsted base character of the 2VP units forming pyridium salts in the presence of the tetrachloroauric acid (HAuCl_4_) through the protonation of the pyridine moieties.^23,50^ Upon the selective gold salt impregnation into the P2VP domains, an enhanced electronic contrast between the PS and P2VP layers is obtained due to the higher electronic density of the P2VP + Au lamellae (therefore, appearing bright in backscattering SEM image mode) while the BCP morphology is preserved (see Fig. 1B). As shown on the AFM views in Fig. S2B,† this step results in a topographical contrast inversion between the PS (light) and the P2VP (dark) domains due to the swelling of the P2VP domains by the gold salts.

An O_2_ RIE plasma was then applied to reduce the Au salts sequestered in the P2VP domains into Au(0).^46,51^ For short exposure times (O_2_ 10 sccm, 60 W, 10 s), the plasma treatment results in the formation of small gold dots within the P2VP domains, as well as the partial removal of the P2VP material (see Fig. 1C and S2C†). Interestingly,^52^ the plasma resistant PS lamellae remain, so that polymer walls are observed between the gold-rich domains. When the plasma exposure is longer (O_2_ 10 sccm, 60 W, 60 s), it results in the complete removal of the BCP thin film and the growth of individualized gold NPs along the initial P2VP lamellae (see Fig. 1D and S2D†).

Consequently, tuning the plasma treatment affords an additional lever for the production of complex plasmonic surfaces as both Au dots confined between PS walls and individualized Au dots were produced as shown in Fig. 1C and D, respectively (see also Fig. S2C and D†).

**Fig. 3:**
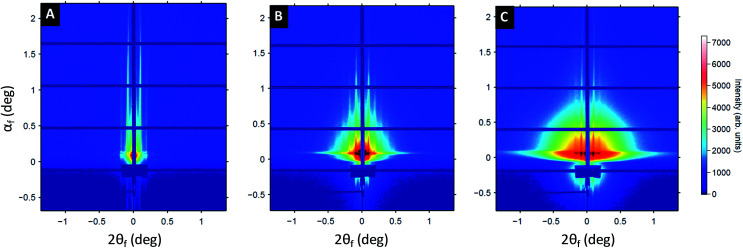
2D GISAXS patterns performed at different steps of the fabrication process of gold decorated surfaces. (A) After the BCP thin film deposition. (B) After the selective incorporation of the gold salts into the P2VP domains. (C) After the reduction of the gold precursors into Au(0) by using an O_2_ RIE plasma treatment (10 sccm, 60 W, 60 s).

To gain further insight on the formation of the Au plasmonic surfaces, GISAXS measurements were performed at different stages of the process (see Fig. 3). At the first step of the process, the 2D GISAXS pattern consists of intense Bragg rods related to the out-of-plane orientation of the periodic BCP lamellae spontaneously formed during the spin-coating process. The GISAXS pattern line-cut along *q*_*y*_ integrated around the Yoneda band, (see Fig. S3†), confirmed the lamellar structure for the as-cast BCP thin film since a first Bragg rod maximum at q* = 0.103 nm^−1^ (*L*_0_ = 2π/q* = 61 nm, in accordance with the AFM and SEM images) and higher order Bragg rods at *q*/q* = 2 and 3 are clearly visible (see Fig. 3A). The hybridization of the out-of-plane P2VP lamellae with the Au salts results in a strong enhancement of the scattering contrast, as shown in Fig. 3B, due to the high scattering cross-section of Au atoms. Higher-order Bragg rods appear (up to the 5^th^ order), but the lamellar signature remains otherwise unchanged (*L*_0_ = 61 nm, see Fig. S3B†). At the last stage of the process, the same sequence of Bragg rods is still observed, although the 2D GISAXS pattern is now dominated by a signal attributed to the form factor of the metallic dots obtained after complete reduction of the gold salts and removal of the BCP template by plasma etching (see Fig. 3C and S3C†). This allows us to conclude on a good transfer of the BCP template dimensions over large surfaces during the fabrication process.

**Fig. 4:**
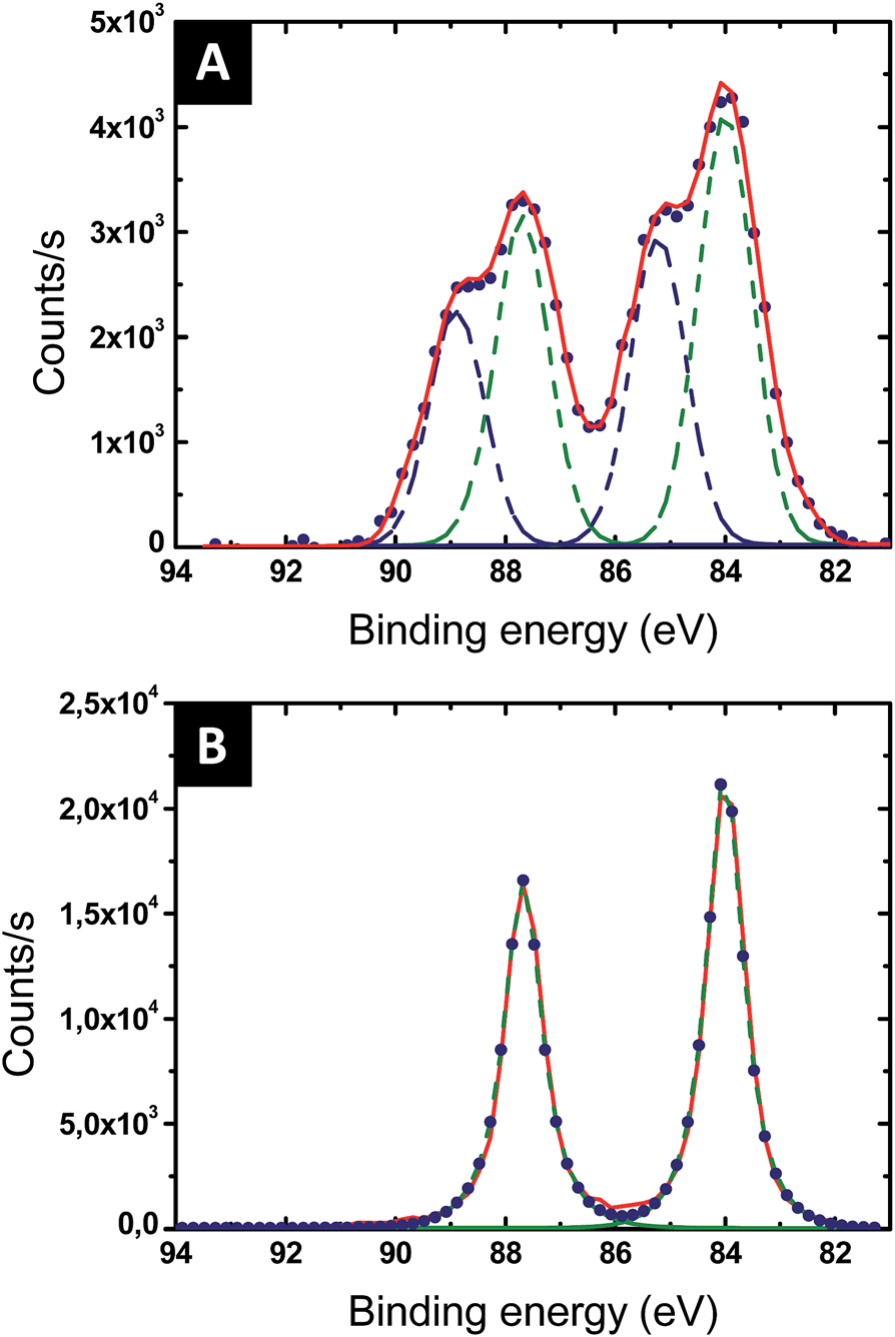
High resolution XPS of Au 4f: blue dots display the experimental measurements, dashed blue and green lines are the pure compound signals and the red full line is the sum of the several dashed lines signals. (A) After impregnation with the gold salt. This profile is indicative of a mixture of Au(i) and Au(iii). (B) After reduction of Au ions *via* 60 s O_2_ plasma treatment. This profile indicates Au(0) exclusively.

The reduction of the Au salts sequestered within the P2VP domains into metallic gold was followed by XPS. The Au 4f XPS spectrum of the BCP thin film loaded with the Au salts is presented in Fig. 4A. Two chemical gold states are detected, corresponding to Au(iii) (90.6 and 87.0 eV – blue dotted line in Fig. 4A) and to Au(i) (88.2 and 84.8 eV – green dotted line in Fig. 4A). The amount of Au(i) is significant, suggesting that the gold salts were already partially reduced by either the contact with the pyridine moieties or the effect of ambient light. After 60 s of plasma treatment, the Au 4f XPS spectrum is consistent with Au(0) since only the characteristic binding energies of 87.6 eV and 83.9 eV are observed (see Fig. 4B).^46^

Further evidence of the Au salts reduction was obtained by KPFM by measuring the contact potential difference (CPD), *V*_CPD_, which is related to the difference between the work functions of the sample *W*_sample_ and the tip *W*_tip_:2
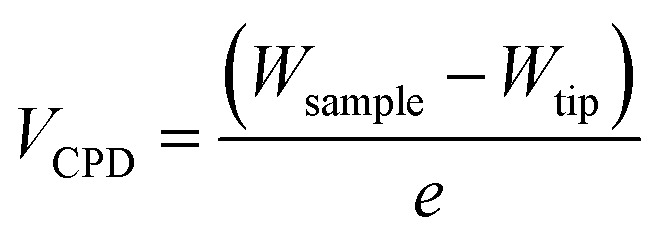
where *e* is the elementary charge.

**Fig. 5:**
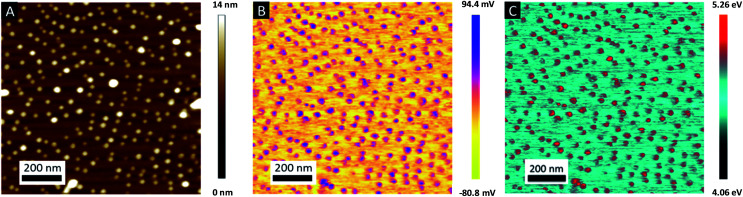
(A) Topography and (B) CDP images obtained using the frequency modulation mode of KPFM on gold NPs on Si substrate after 60 s of O_2_ plasma. (C) Work function map (in eV) of the sample surface retrieved from the composite topographical and CPD images.

The topographic view of the surface decorated by the metallic dots produced after a complete removal of the BCP template presented in Fig. 5A is consistent with its associated CPD image (see Fig. 5B), with a significantly lower *V*_CPD_ value for the SiO_2_ substrate (*V*_CPD_ = 0.6 V) than for the gold NPs (*V*_CPD_ = 1.3 V) (see Fig. S4†). Considering a work function of 4.09 eV for PFQNE-AL tip^53,54^ the work function of gold NPs and SiO_2_ substrate can be estimated, according to eqn (2), to 4.67 eV for SiO_2_ and 5.37 eV for Au. These estimates are in good agreement with the theoretical work functions^55^ for SiO_2_ (*W*_SiO_2__ = 4.3 eV) and Au (*W*_Au_ = 5.3 eV) (see Fig. 5C).

UV irradiation, known for triggering the photodecomposition of HAuCl_4_ precursors into Au(0) within gold-loaded polymer nanocomposite thin films^45,56^ was used as an alternative to the RIE plasma in order to preserve the BCP template (see Fig. S5 and S6†). TEM images of the gold-loaded polymer nanocomposite thin films exposed for 6, 24 and 48 h under UV light (*λ* = 254 nm, 6 W) are shown in Fig. S5.† The results demonstrate that an additional lever is provided by this alternative methodology as gold NPs with different sizes and shapes are produced by varying the UV irradiation exposure duration. After 6 h of exposure, the TEM image shows ill-defined gold agglomerates localized within the P2VP lamellar domains (Fig. S5B†). Further exposure to UV light (see Fig. S5C and D†) leads to larger nanoparticles with a size distribution centred on 7 nm after 24 h of exposure (see Fig. S5C and E†) and a bimodal size distribution centred on 10 and 15 nm after 48 h of exposure (Fig. S5D and F†). It is noteworthy that 24 h of UV light exposure is effective to fully reduce the gold salts as confirmed by the XPS spectrum presented in Fig. S6.†

**Fig. 6:**
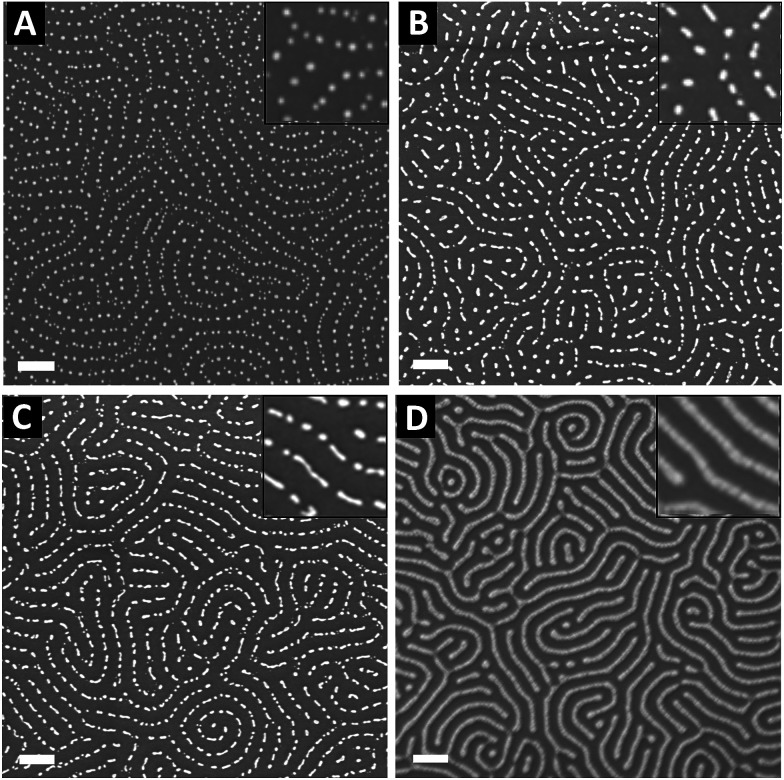
SEM images of discreet gold nanoparticles arrays formed on a silicon substrate using a PS-*b*-P2VP BCP template by immersion in a 1 wt% aqueous gold precursor solution for different times and a subsequent O_2_ RIE treatment. (A) 1 h, (B) 48 h and (C) 120 h. (D) SEM image of continuous gold lines formed on a silicon substrate after 120 h of immersion in the gold precursor solution and a subsequent Ar plasma treatment. Scale bars = 100 nm.

To control the optical properties of the nanostructured plasmonic surfaces, the shape of the NPs was tuned by varying the metal content within the P2VP lamellae. To this purpose, the PS-*b*-P2VP thin films were immersed in a HAuCl_4_ aqueous solution for different immersion durations in order to vary the amount of gold precursors incorporated into the P2VP lamellae. Importantly, water was chosen as the solvent to avoid surface reconstruction of the BCP thin film due the swelling of the P2VP domains observed when using ethanol.^57,58^ SEM images presented in Fig. 6 show the panel of plasma etched surfaces having different gold nanofeature shapes produced by varying the immersion duration from 1 h to 120 h. After 1 h of immersion followed by an O_2_ RIE plasma, well-defined Au dots with a 20 nm diameter were produced (see Fig. 6A), while rod-like Au particles are formed when the immersion duration reached 48 h (see Fig. 6B). By further increasing the immersion time to 120 h, an increase of the length of the Au rods is observed, leading to the formation of Au dashed lines on the SiO_2_ surface as shown in Fig. 6C. For the same immersion duration (*i*.*e*., 120 h), it turns out that continuous gold lines are produced when an Ar plasma (Ar 10 sccm, 60 W, 60 s) is used to reduce the gold precursors (see Fig. 6D).

**Fig. 7:**
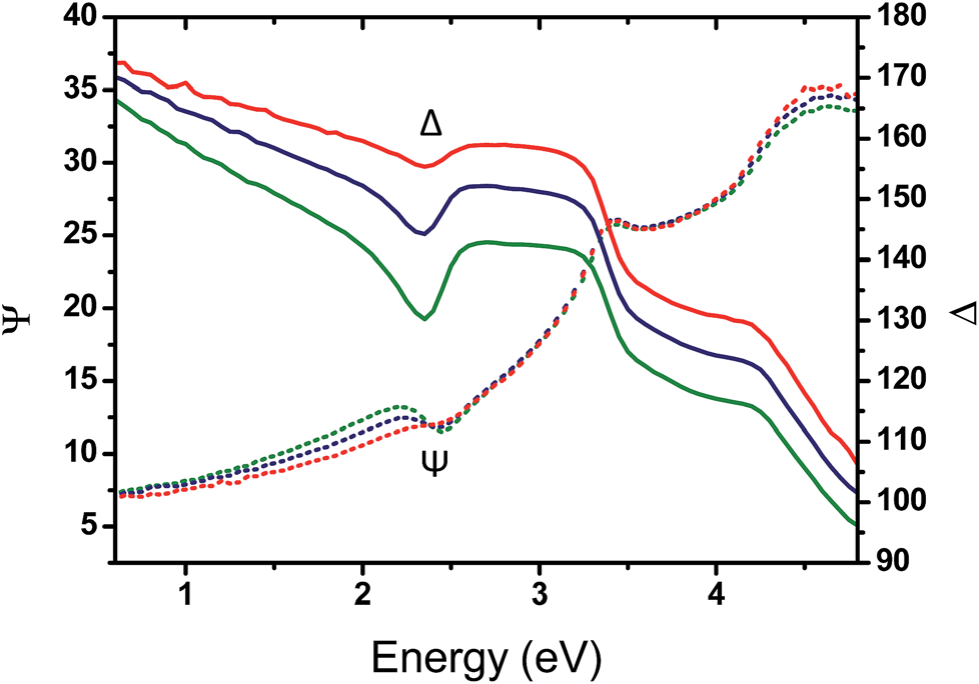
Evolution of the measured ellipsometric angles, *Ψ* (full lines) and *Δ* (dotted lines), as a function of the photon energy for an angle of incidence of *θ* = 70° for three different immersion times 1 h (red), 48 h (blue) and 120 h (green).

To gain insight into the effect of the gold nanofeature shape on the surface plasmonic properties, key samples (*i.e.*, immersed for 1 h, 48 h and 120 h in an HAuCl_4_ aqueous solution followed by an O_2_ plasma treatment) were further analysed by VASE. The evolution of the measured ellipsometric angles *Ψ* and *Δ* as a function of the photon energy, between 0.6 and 4.8 eV, reveals a clear resonance feature in the region near 2.3 eV, which is attributed to the effect of the LSPR of the Au NPs templated from the P2VP domains (see Fig. 7).

A progressive evolution of the resonance band is observed, due to the increase of the gold amount on the surface and the shape evolution of the produced Au NPs. A slight shift in the SPR spectral position is also observed, from 2.30 eV (539 nm) to 2.20 eV (563 nm).

Extracting, from the ellipsometric data, the optical index *ñ* = *n* + i*k* or the permittivity *ε* = *ε*_r_ + i*ε*_i_ of a thin film of material deposited on a substrate requires on the one hand a multilayer ellipsometric model representing the sample + substrate system, whereby indices and thicknesses of most layers are known, and on the other hand, an appropriate optical model of the film, which can be challenging for nanostructured and anisotropic materials, including some degree of disorder, such as the ones studied here. The studied films are represented by a multilayer ellipsometric model comprising a silicon semi-infinite substrate, a first layer of SiO_2_ of thickness 2 nm and the unknown (sample) film of thickness *t*. In a first approximation, nanocomposite materials can be represented with a simple effective medium law, which relates the effective permittivity of the composite medium to the permittivities of the constituent materials, irrespective of the precise structure of the composite provided that the characteristic dimensions are smaller than the wavelength of the incident light. The simplest effective medium law is the Maxwell Garnett formula,^59^ which is well adapted for dilute spherical inclusions in a matrix and is given by:3
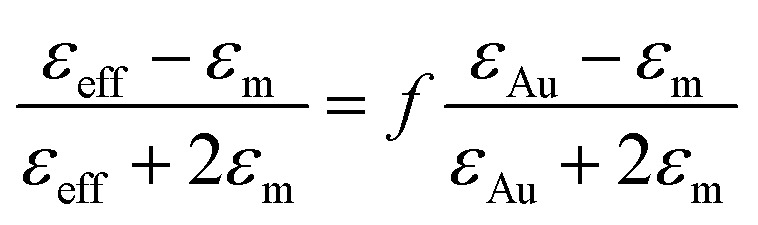
where *ε*_eff_ is the effective permittivity of the gold NP–air composite, *ε*_m_ is the matrix permittivity, here for air *ε* = 1, *ε*_Au_ is the NP gold permittivity, and *f* is the gold volume fraction in the film.

**Fig. 8:**
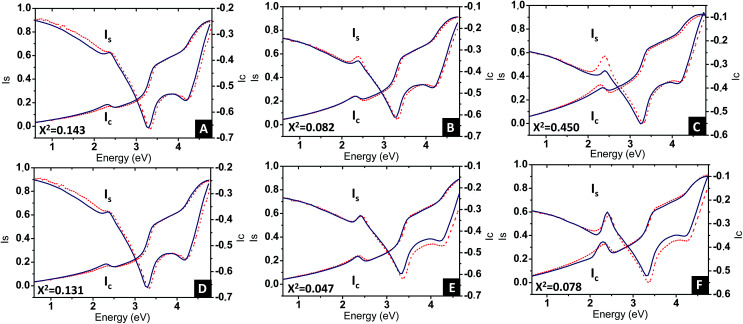
Plots of the experimental ellipsometric quantities, *I*_s_ and *I*_c_ (red dotted curves) and the corresponding fitting using (A–C) spherical Maxwell Garnett formula and (D–F) non-spherical Maxwell Garnet formula (blue continuous lines), for three different immersion times (A, D) 1 h, (B, E) 48 h and (C, F) 120 h. In each graph is given the value of the *χ*^2^ parameter, indicative of the goodness of the fits.

Eqn (3) was applied to the set of measured *I*_s_ and *I*_c_ values over all measured angles of incidence for each sample at different immersion times (1, 48 and 120 h), using for the gold permittivity a function modified from the Johnson & Christy tabulated data.^60,61^ The plots of the experimental results, for the incident angle 70°, and the corresponding fits are presented in Fig. 8A–C.

Two fitting parameters are used: the gold volume fraction *f* (involved in eqn (1)), and the thickness, *t*, of the film (involved in the ellipsometric model). Fitting is satisfactory for the spectra in the cases of short immersion times when the produced NPs are spherical (Fig. 8A). On the opposite, when the NPs are elongated, the quality of the fits decreases, particularly in the spectral region of the LSPR of the gold nanoparticles (≈2.4 eV) (Fig. 8B and C). In order to obtain better-quality fits, we used a modified Maxwell Garnett formula dedicated to disordered ellipsoids^62,63^ defined as:4
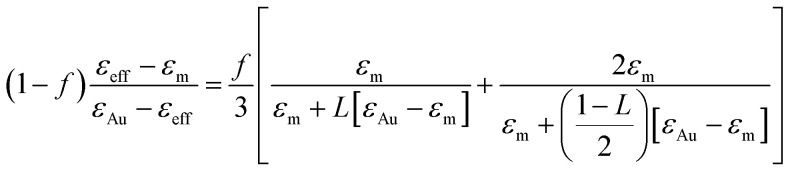


This function applies to an assembly of ellipsoids of revolution (or spheroids) of dimensions *a* = *c* ≠ *b*, with *a*, *b* and *c* the three principal semi-axes of the ellipsoids and *L* the depolarization factor given by:5
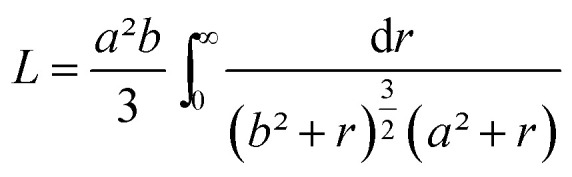
*L* is related to the aspect ratio, *b*/*a*, of the ellipsoids, and values for *L* with 0 ≤ *L* ≤ 1/3 (prolate ellipsoids), *L* = 1/3 (sphere) and 1/3 ≤ *L* ≤ 1 (oblate ellipsoids) are possible.

Using this effective medium law, three parameters were fitted, the gold volume fraction *f*, the depolarization factor *L*, and the film thickness *t*. Eqn (4) was applied to the set of measured *I*_s_ and *I*_c_ values over all measured angles of incidence for each sample at different immersion times (1, 48 and 120 h). The plots of the experimental results, for the incident angle 70°, and the corresponding fittings are presented on Fig. 8D–F. The fit quality is significantly improved, especially around the LSPR of the gold NPs. The resulting structural fitting parameters are presented in Table 1, in which is also reported the height of the gold NPs measured on the profiles of the AFM topographic images. Both extracted values are in very good agreement, which underlines the robustness of the treatment. The values of *L* extracted from the ellipsometry data fits, for samples with immersion times 48 and 120 h, are smaller than 1/3 and therefore indicate prolate ellipsoids, as expected from the elongated shapes visible on the SEM images. We can then extract the corresponding aspect ratio, *b*/*a*, using the equation:6
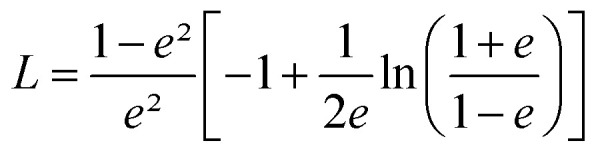
with 
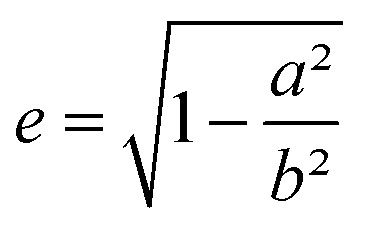
 the eccentricity of the ellipsoids.

**Table 1:** Sample structural parameters with *t*_MG_ the film thickness extracted from the fit of the ellipsometry data to the modified Maxwell Garnett formula, *t*_AFM_ the film thickness obtained from the AFM topographical profiles. *f*_MG_ the gold volume fraction and *L*_MG_ the depolarization factor are both extracted from the fit of the ellipsometry data to the modified Maxwell Garnett formula (eqn (4)). From *L*_MG_, we can extract an aspect ratio, *b*/*a*_MG_, and compare it to the mean aspect ratio *b*/*a*_SEM_ given by statistical analysis of the SEM images

Immersion time	*t*_MG_ (nm)	*t*_AFM_ (nm)	*f*_MG_ (%)	*L*_MG_	*b*/*a*_MG_	*b*/*a*_SEM_
1 h	8	8.5	5.1	0.3	1.1	1.1
48 h	9.3	9.7	10.3	0.23	1.5	1.4
120 h	10.1	10.4	15.7	0.18	1.9	2

Figures S7–S9† present the aspect ratio obtained by analysing the SEM images of the Au structures obtained. All parameters are listed in Table 1. The good agreement of the ellipsoidal Maxwell Garnett model, applying to non-interacting NPs, with both the ellipsometric data and the structural analysis indicates that plasmonic couplings are not significant in the optical response of the nanostructured BCP films.

**Fig. 9:**
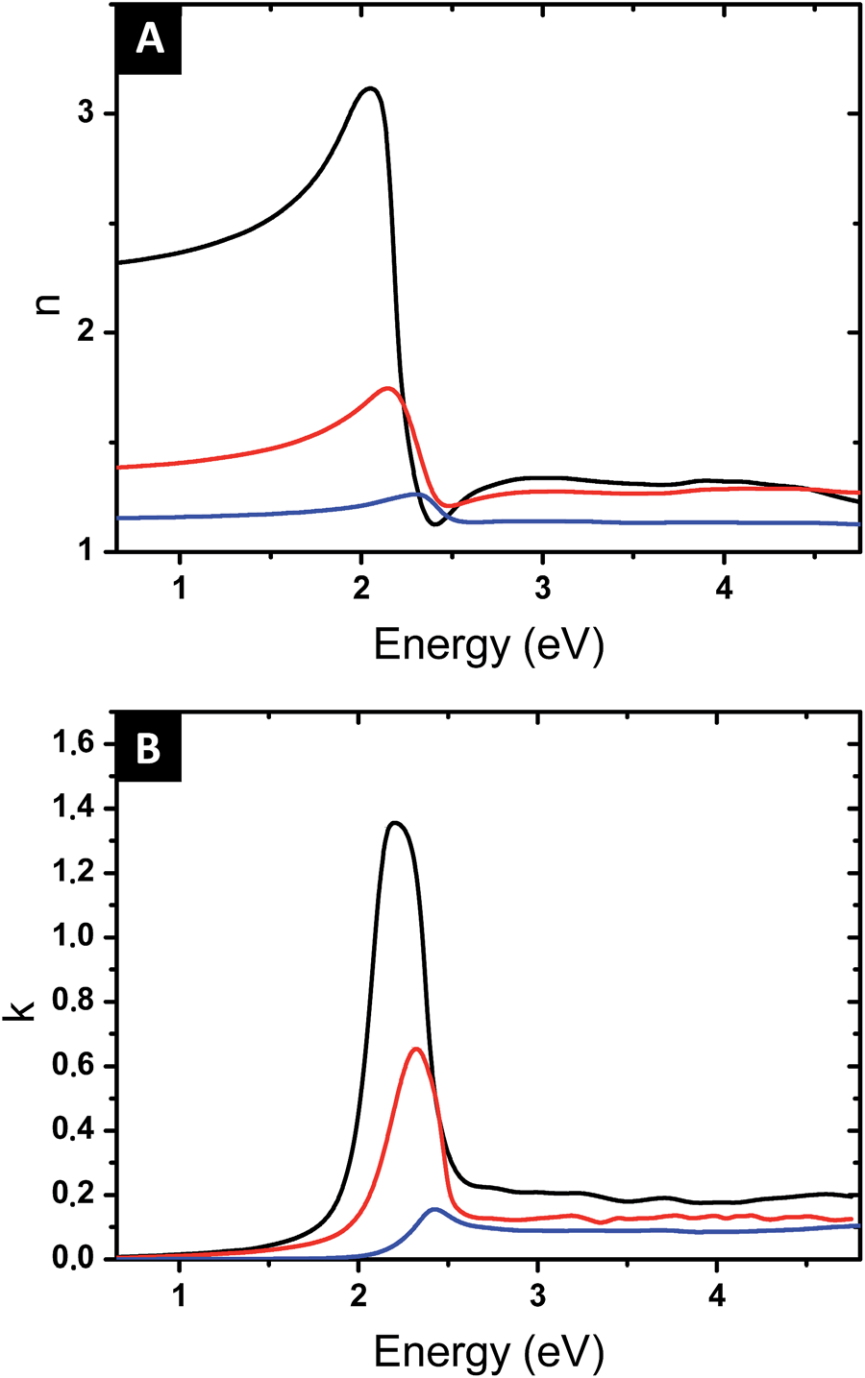
Optical index of the studied nanostructured surfaces obtained by the modified Maxwell Garnett formula. The blue, red and black curves correspond to the samples obtained from immersion time of 1 h, 48 h and 120 h respectively.

Once the parameters *f* and *L* have been determined, the Maxwell Garnett formula (eqn (4)) gives access to the optical index of the composite film. Fig. 9 shows the values of *n* and *k*, as a function of the photon energy, for the samples with immersion times 1, 48 and 120 h. All films present a resonant behaviour, but the amplitude of this resonance greatly increases with immersion time, and its spectral position shifts to lower energy, from 506 to 576 nm in wavelength. These evolutions are related to the controlled increment in the gold volume fraction and the increased aspect ratio of the elongated produced NPs. The precise control on the shape, structure and volume fraction obtained with the fabrication process, along with the effect of the localized surface plasmon, allows modulating the optical response while keeping a low gold content and little plasmonic coupling effects.

High-refractive index surfaces (∼3.2) with relatively low extinction coefficient (1.4) are obtained at the maximum time of immersion (120 h). It is useful to note that an application of the simple spherical Maxwell Garnett model with similar gold volume fraction values would provide significantly lower values of *n* (*n*_max_ = 1.4 *vs.* 1.8 for 10.3 vol% and *n*_max_ = 1.6 *vs.* 3.2 for 15.7 vol%). On the other hand, nanoplasmonic surfaces with *n* values as high as 5 reported in the literature^12^ required a gold content above 40 vol%.

## Conclusions

The production of surfaces with a high and controllable refractive index is a challenge for new optical applications. Indeed, the refractive index of natural transparent materials is an intrinsic material property which is difficult to tailor and normally limited to a value below 2. Here, a straightforward strategy enabling the fabrication of nanostructured plasmonic surfaces with a high refractive index is demonstrated by using block copolymer thin films as regular patterned templates. Reproducible spherical and rod-like gold NP arrays over large surface area were produced by varying the surface preparation parameters, which allows tailoring the material refractive index. The effective optical properties of this type of samples were reproduced by a simple Maxwell Garnett model, adapted for non-spherical objects. Finally, high refractive index values were obtained, due to the nanoparticle shape rather than plasmonic couplings.

## Conflicts of interest

There are no conflicts to declare.

## Supplementary Material

NA-001-C8NA00239H-s001
