# Supplementary material for: High refractive index in low metal content nanoplasmonic surfaces from self-assembled block copolymer thin films
Source: Nanoscale Adv. 2018 Nov 22;1(2):849–57. doi: 10.1039/c8na00239h (PMC9473184; doi:10.1039/c8na00239h)
Supplement: NA-001-C8NA00239H-s001 [file NA-001-C8NA00239H-s001.pdf]

Electronic Supplementary Material (ESI) for Nanoscale Advances.
This journal is © The Royal Society of Chemistry 2018

SUPPORTING INFORMATION

High refractive index in low metal content nanoplasmonic surfaces from self-assembled block copolymer thin films

Alberto Alvarez-Fernandez,[a,b] Karim Aissou,[a] Gilles Pécastaings,[a] Georges Hadziioannou,[a] Guillaume Fleury*[a] and Virginie Ponsinet*[b]

[a]Laboratoire de Chimie des Polymères Organiques (LCPO), CNRS UMR 5629 - ENSCPB - Université de Bordeaux, 16 Avenue Pey-Berland, F-33607 Pessac Cedex, France

[b]Univ. Bordeaux, CNRS, Centre de Recherche Paul Pascal (CRPP), UMR 5031, 115 avenue Schweitzer, F-33600 Pessac, France

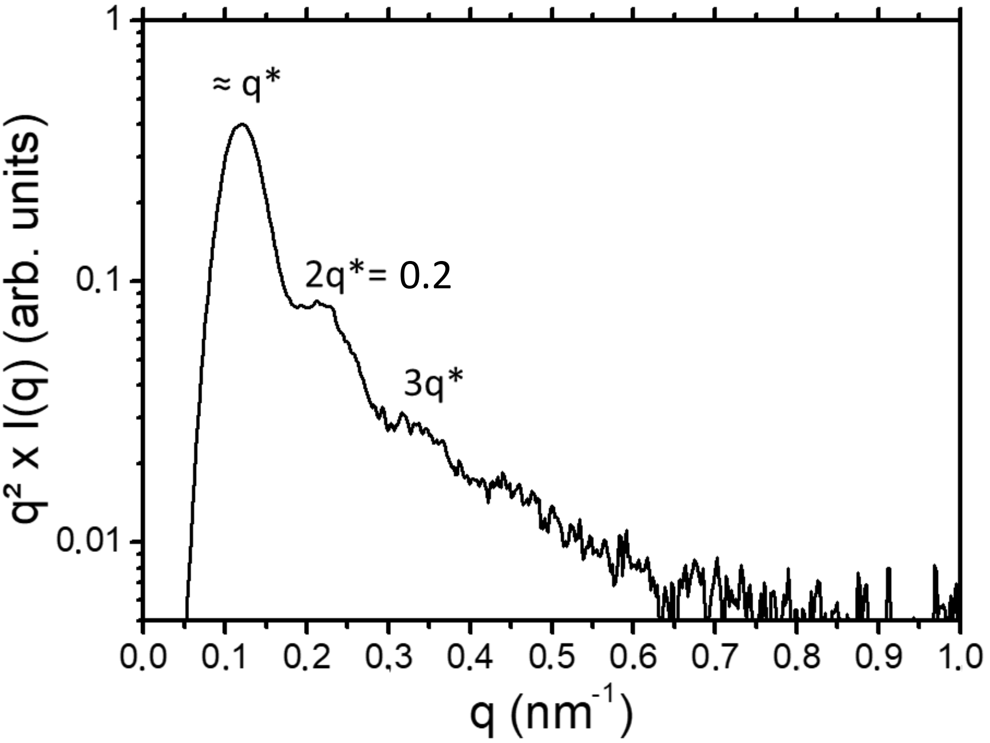

Figure S1. SAXS signature, plotted as $q^2I(q)$ versus q, with q the scattering wavevector and I(q) the scattered intensity of a bulk sample of the PS-*b*-P2VP copolymer used in this study. The lamellar structure is identified by the successive positions of the peaks at q*, 2q*, 3q*. The SAXS intensity is multiplied through by q^2, as a first-order correction for the impact of the form factor P(q) of individual lamellae. The lamellar period is found to be $L_o=4\pi/(2q^*)$ = 63nm.

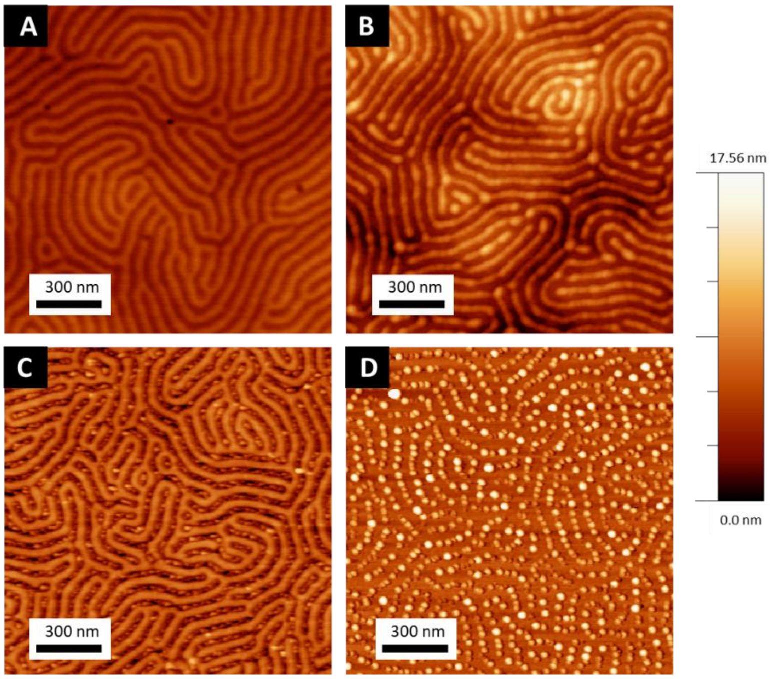

Figure S2. AFM topographical views of (A) the out-of-plane PS-*b*-P2VP lamellar structure obtained after spin-coating, (B) the gold-loaded structure produced by spin-coating a 1 wt% gold precursor solution on top of the nanostructured block copolymer film, and (C) and (D) the arrays of gold nanoparticles obtained after (C) 10 s and (D) 60 s of O_2 RIE plasma treatment, respectively. These are the AFM equivalent of the SEM micrographs shown in the Figure 2.

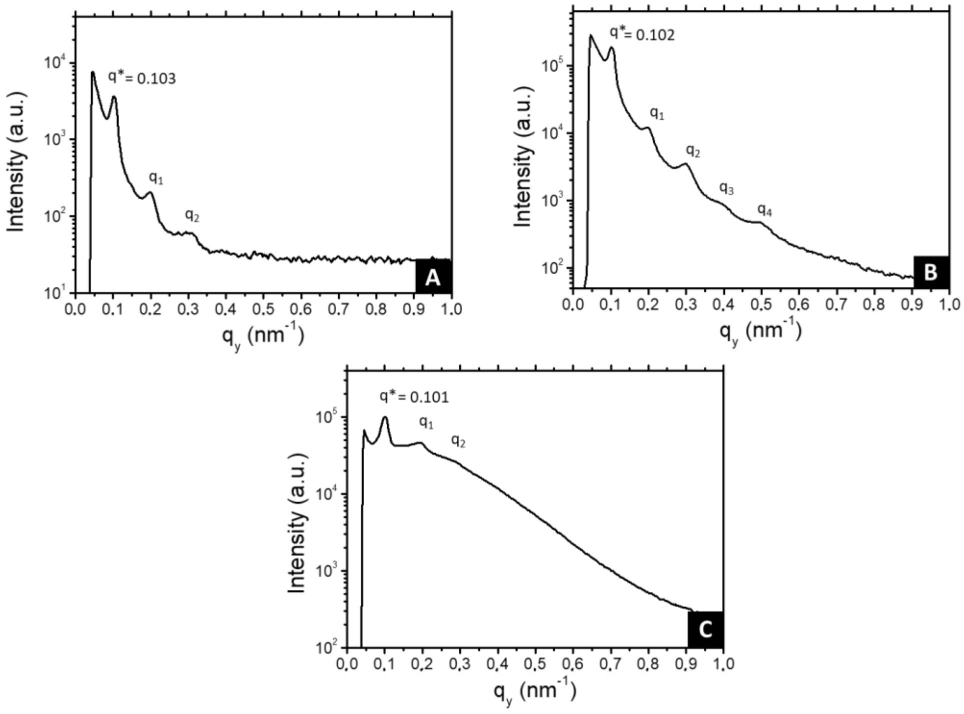

Figure S3. GISAXS pattern line-cut along q_y integrated around the Yoneda band of the film at different steps of the process, (A) after polymer deposition, (B) with the gold salt selectively incorporated to the P2VP domains and (C) after 60s of O_2 RIE.

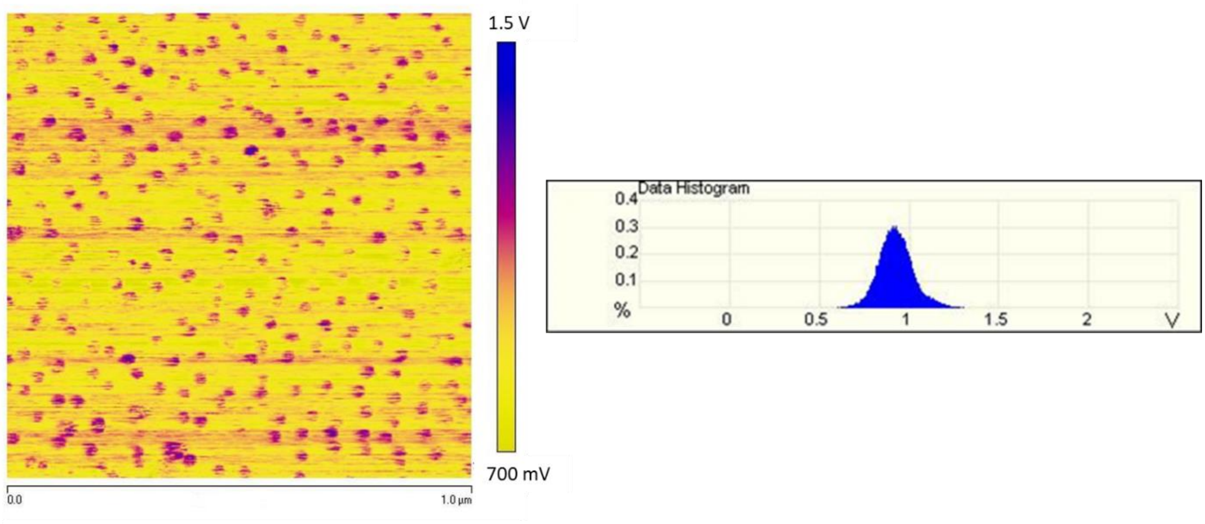

Figure S4. KPFM surface potential without flattening with the histogram of the measured potential.

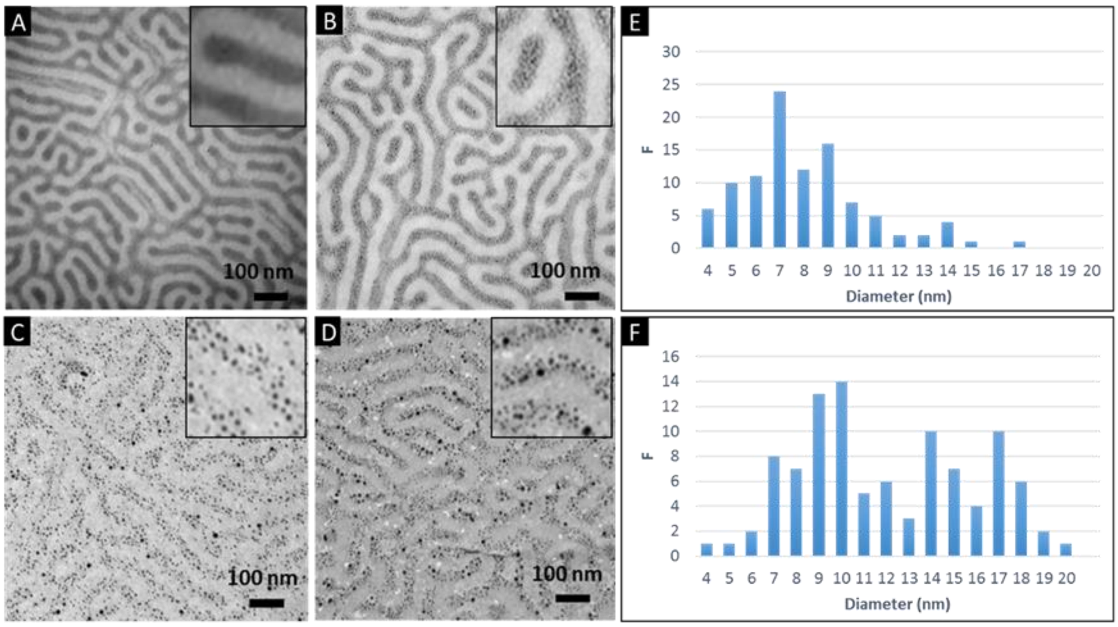

Figure S5. TEM micrographs of the gold/polymer composite surfaces at different times of UV irradiation. (A) Neat block copolymer film, (B) 6h UV irradiation, (C) 24h UV irradiation and (D) 48h UV irradiation. Size distribution histograms of the gold NPs, obtained from the micrographs of the (E) 24h and (F) 48h irradiated samples.

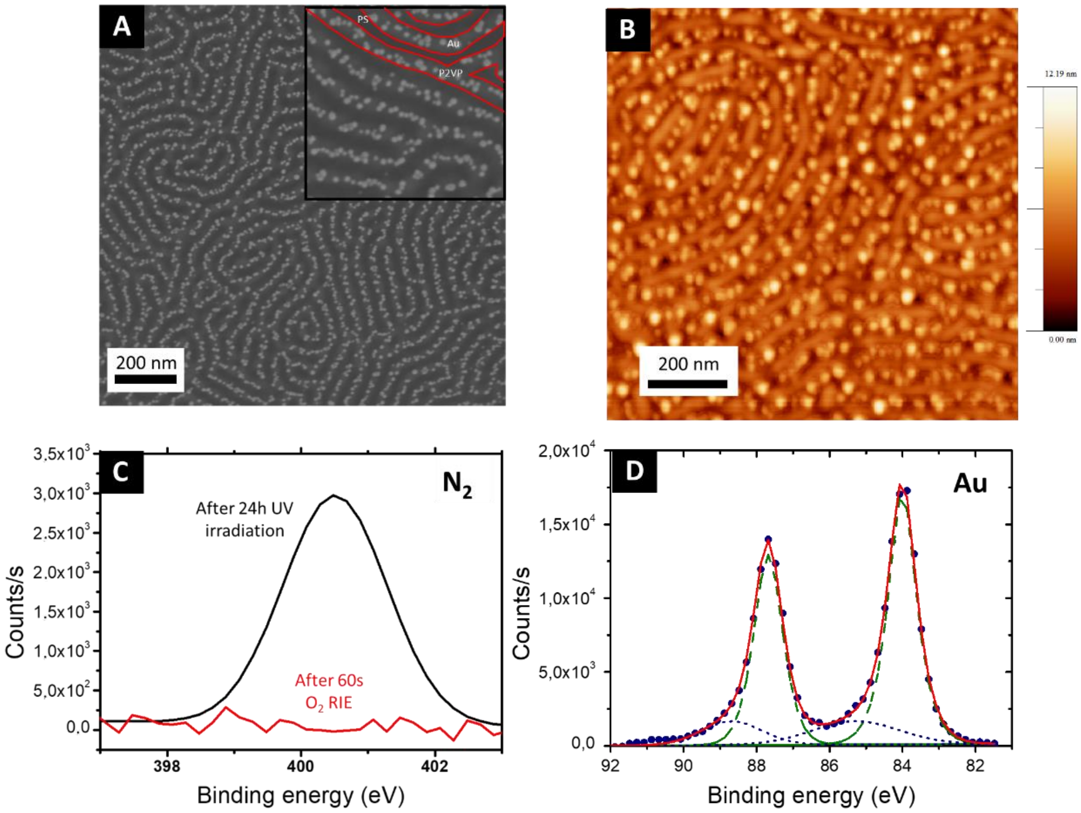

Figure S6. (A) SEM, (B) AFM topographic images and the corresponding (C) N_2 and (D) Au XPS spectrum of a PS-*b*-P2VP sample immersed in the gold precursor solution and exposed to UV irradiation for 24h. A three-color pattern attributed to the PS, P2VP and Au can be observed in the SEM image. The AFM micrograph and N_2 XPS spectrum confirm that the polymer film has not been damaged during the exposure. For comparison the N_2 XPS spectrum of PS-*b*-P2VP sample immersed in the gold precursor and exposed to 60s of O_2 RIE is provided. The Au XPS spectrum confirms the gold reduction during the UV irradiation.

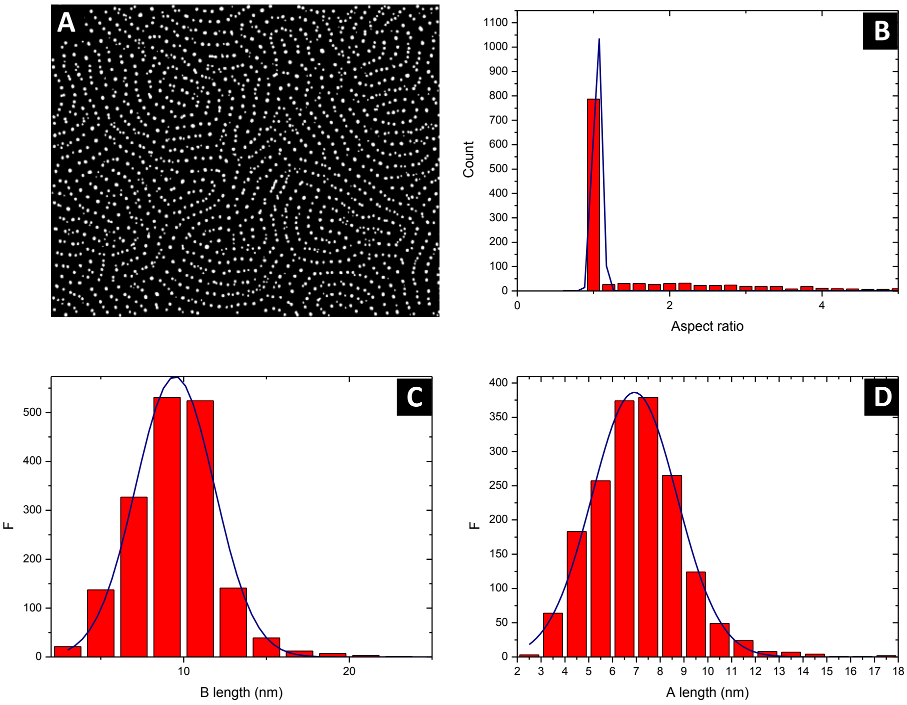

Figure S7. (A) SEM image after 1h of immersion converted to a B/N 8 bits image and treated with the Visio Assistant software. (B) Histogram of the aspect ratio, and (C and D) histograms of dimensions of the particles, all fitted with Gaussian distributions.

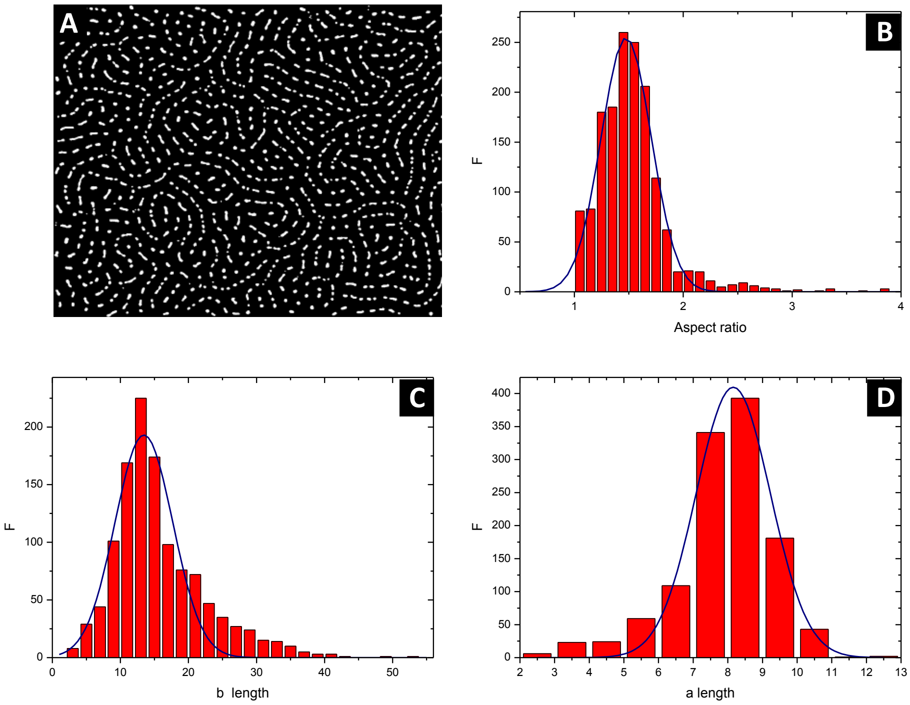

Figure S8. (A) SEM image after 48h of immersion converted to a B/N 8 bits image and treated with the Visio Assistant software. (B) Histogram of the aspect ratio, and (C and D) histograms of dimensions of the particles, all fitted with Gaussian distributions.

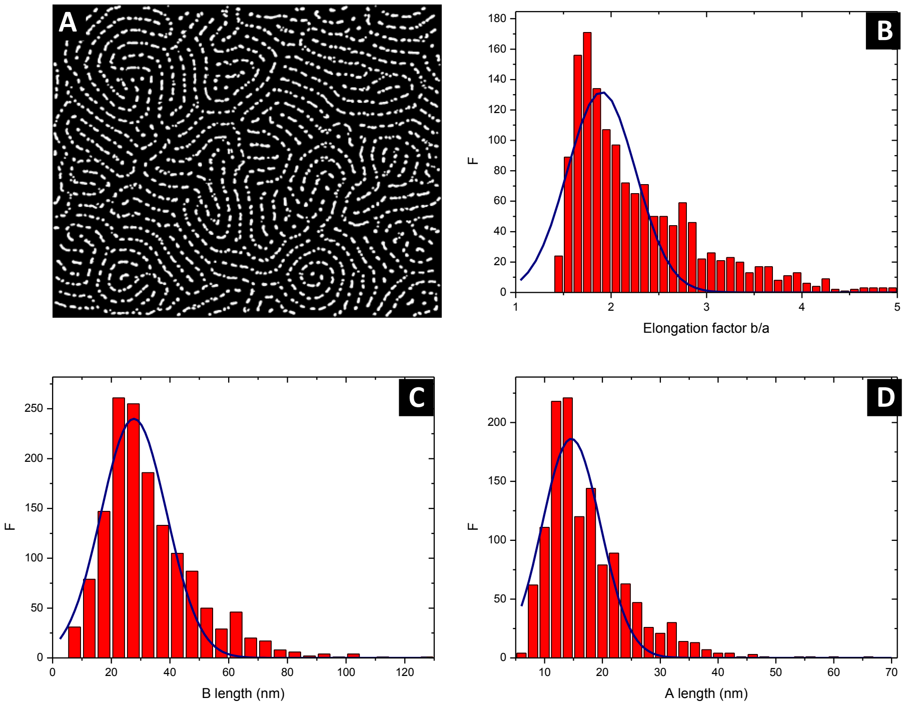

Figure S9. (A) SEM image after 120h of immersion converted to a B/N 8 bits image and treated with the Visio Assistant software. (B) Histogram of the aspect ratio, and (C and D) histograms of dimensions of the particles, all fitted with Gaussian distributions.
